# Hypersensitivity Reactions to Selpercatinib Treatment With or Without Prior Immune Checkpoint Inhibitor Therapy in Patients With NSCLC in LIBRETTO-001

**DOI:** 10.1016/j.jtho.2022.02.004

**Published:** 2022-02-17

**Authors:** Caroline E. McCoach, Christian Rolfo, Alexander Drilon, Mario Lacouture, Benjamin Besse, Koichi Goto, Viola W. Zhu, Daniel S. W. Tan, Stephanie Farajian, Laura A. Potter, Jennifer F. Kherani, Victoria Soldatenkova, Elizabeth A. Olek, Catherine E. Muehlenbein, Keunchil Park

**Affiliations:** a Helen Diller Family Comprehensive Cancer Center, University of California San Francisco, San Francisco, California; b Present Address: Genentech, Inc., South San Francisco, California; c Greenebaum Comprehensive Cancer Center, Experimental Therapeutics Program, School of Medicine, University of Maryland, Baltimore, Maryland; d Present Address: Center for Thoracic Oncology at Tisch Cancer Institute, Mount Sinai Health System and Icahn School of Medicine at Mount Sinai, New York, New York; e Memorial Sloan Kettering Cancer Center and Weill Cornell Medical College, New York, New York; f Department of Cancer Medicine, Gustave Roussy Cancer Campus, Villejuif, France; g Department of Thoracic Oncology, National Cancer Center Hospital East, Kashiwa, Japan; h Department of Medicine, Division of Hematology and Oncology, University of California Irvine, Orange, California; i National Cancer Centre Singapore, Duke-National University of Singapore Medical School, Singapore; j Present Address: Davis School of Medicine, University of California, Sacramento, Sacramento, California; k Loxo Oncology, Inc., a wholly owned subsidiary of Eli Lilly and Company, Stamford, Connecticut; l Eli Lilly and Company, Indianapolis, Indiana; m Samsung Medical Center, Sungkyunkwan University School of Medicine, Seoul, Republic of Korea

**Keywords:** Hypersensitivity, Selpercatinib, Immune checkpoint inhibitor, Non–small-cell lung cancer, Supportive care

## Abstract

**Introduction:**

Immune checkpoint inhibitor (ICI) therapy has been found to increase the risk/severity of immune-mediated adverse events with subsequent kinase inhibitor treatment in oncogenically driven cancers. We explored the risk for hypersensitivity with selpercatinib, a first-in-class highly selective and potent, central nervous system-active RET inhibitor, in prior ICI-treated patients with *RET* fusion-positive NSCLC compared with their ICI-naive counterparts.

**Methods:**

Data from patients enrolled by December 16, 2019, in the ongoing phase 1/2 LIBRETTO-001 (NCT03157128) trial were analyzed for hypersensitivity reactions reported using preferred terms of hypersensitivity/drug hypersensitivity and defined as a constellation of symptoms/findings characterized by maculopapular rash, often preceded by fever with arthralgias/myalgias, followed by greater than or equal to 1 of the following signs/symptoms: thrombocytopenia, increased aspartate aminotransferase or alanine aminotransferase, hypotension, tachycardia, or increased creatinine.

**Results:**

Of 329 patients, 22 (7%) who experienced a grade 1 to 3 hypersensitivity reaction that met the defined constellation of events were attributed to selpercatinib by investigators, and more often in prior ICI-treated (n = 17, 77%) than ICI-naive (n = 5, 23%) patients. There were 19 patients with selpercatinib-related hypersensitivity who resumed selpercatinib post-hypersensitivity with dose modification/supportive care. Furthermore, 17 patients, of whom 14 received prior ICI therapy, were still on treatment at twice daily doses of 40 mg (n = 5), 80 mg (n = 4), 120 mg (n = 4), and 160 mg (n = 4).

**Conclusions:**

Rates of selpercatinib-related hypersensitivity were low overall and, as with other kinase inhibitors, occurred predominantly in prior ICI-treated patients. Hypersensitivity to selpercatinib can be managed with supportive care measures regardless of prior ICI status and is reversible.

## Introduction

*RET* gene alterations are oncogenic drivers of several cancer types.^1^ *RET* fusions are present in about 1% to 2% of patients with NSCLC.^2–4^ Selpercatinib is a first-in-class highly selective and potent RET kinase inhibitor with central nervous system activity. Selpercatinib was found to have robust and durable efficacy with a favorable safety profile in patients with advanced or metastatic RET-driven, treatment-naive, and previously treated cancers, regardless of prior therapy.^5,6^ Notable activity was achieved in treatment-naive patients.^5,6^ As a result, selpercatinib is approved in multiple countries for the treatment of RET-altered lung or thyroid cancers.^7,8^

We previously reported that treatment-emergent hypersensitivity reactions from any cause occurred in 30 of 702 (4%) selpercatinib-treated patients with *RET*-positive solid tumors in LIBRETTO-001.^7^ Of the 30 cases reported in the overall population, the proportion of patients who experienced a hypersensitivity reaction was highest among patients with *RET* fusion-positive NSCLC (25 of 329 patients, 8%).^9^ Published reports of immune-related reactions to kinase inhibitors in patients previously treated with immune checkpoint inhibitor (ICI) therapy suggest that immunotherapy may increase the risk for and the severity of immune-mediated hypersensitivity reactions with subsequent kinase inhibitor therapy.^10–16^

A detailed characterization of drug-associated hypersensitivity in the global, multicenter, prospective LIBRETTO-001 trial and the conditioning effect of prior ICI therapy have not been reported. This analysis explored the risk for drug hypersensitivity reactions with selpercatinib treatment in patients with *RET* fusion-positive NSCLC with or without prior ICI therapy.

## Materials and Methods

The study design of the LIBRETTO-001 trial has been reported.^5,6^ In brief, this ongoing, global, multicenter, open-label, phase 1/2 study enrolled adults (or adolescents as young as 12 y of age, where approved) with advanced or metastatic *RET*-altered solid tumors, including *RET* fusion-positive NSCLC. Both treatment-naive and previously treated patients were eligible. Any number of prior treatments were acceptable, including (but not limited to) ICIs, multikinase inhibitors, and chemotherapy. At the discretion of the investigator, intrapatient dose escalation to dose level cohorts previously cleared by the safety review committee was permitted in phase 1, and dose re-escalation after dose reduction owing to an adverse event (AE) was allowed after event resolution in both phases of the trial. The phase 2 recommended selpercatinib dose of 160 mg orally twice daily was administered in consecutive 28-day cycles until disease progression, unacceptable toxicity, death, or withdrawal of consent. The primary end point was objective response rate (ORR) assessed by an independent review committee (IRC) using Response Evaluation Criteria in Solid Tumors version 1.1. Secondary end points included duration of response (DoR), progression-free survival, overall survival, and safety.

In the present analysis, events associated with a drug hypersensitivity reaction were assessed in the overall safety population with *RET* fusion-positive NSCLC enrolled by December 16, 2019. The drug hypersensitivity reaction described herein was included in the Investigator’s Brochure after a review of the initial cases with an oncodermatology specialist. In addition, a study-wide memo was issued to facilitate broad investigator awareness and safety reporting under the unified term of drug hypersensitivity for accurate identification and tracking of future events of a similar nature. Ultimately, subsequent cases were reviewed as reported and the aggregate event details informed the current comprehensive definition: a constellation of symptoms and findings characterized by a maculopapular rash, often preceded by fever with associated arthralgias or myalgias, followed by at least one or more of the following signs and symptoms: more often, thrombocytopenia or increased aspartate aminotransferase (AST) or alanine aminotransferase (ALT), or less often, decreased blood pressure, tachycardia, or increased creatinine. Rash and fever were principal signs/symptoms and, therefore, one or both had to be present (at a minimum) for the AE to be considered a drug hypersensitivity reaction. Severity grading was not predefined. Investigators typically graded severity on the basis of the singular component of the event with the greatest severity (e.g., rash, laboratory abnormality) rather than collective findings. Hypersensitivity reactions graded as 1 (mild) or 2 (moderate) were reported by investigators as serious events if they met one of the protocol-defined criteria (requiring hospital admission, prolongation of an admission, or considered to be a medically important event).

To determine whether prior ICI-treated patients were at higher risk of drug hypersensitivity reactions with selpercatinib treatment, the preferred Medical Dictionary for Regulatory Activities version 21.0 preferred terms “hypersensitivity” and “drug hypersensitivity” were consolidated and assessed by subgroups with or without prior treatment with ICI therapy. Drug-related hypersensitivity was used as a surrogate for this reaction. Hypersensitivity reactions related to factors other than the study drug (e.g., antibiotic therapy [n = 2] and seasonal allergies [n = 1]) were not included.

In addition, treatment-emergent AEs of any type were assessed in the overall safety population and by subgroups with or without prior ICI therapy who were enrolled by December 16, 2019. Tumor response was also measured in patients with drug hypersensitivity and by subgroups with or without prior ICI therapy in the first 105 consecutively enrolled patients who had previously received platinum-based chemotherapy.

## Results

A total of 329 patients with *RET* fusion-positive NSCLC comprised the overall safety population (Table 1). At study enrollment, 152 patients had received prior treatment with ICI therapy, and a total of 177 patients were either naive to systemic treatment (n = 60) or had not received ICI therapy with prior systemic regimens (n = 117). The median ages were 59 years (range: 26–80 y) and 62 years (range: 23–92 y) in prior ICI-treated and ICI-naive subgroups, respectively. Most of the patients in each subgroup were female (52% and 60%) and White (51% and 50%) or Asian (43% and 40%). Patients in the prior ICI therapy subgroup had received more prior lines of systemic therapy (1–2 lines, 47%; ≥3 lines, 53%) than ICI-naive patients (1–2 lines, 48%; ≥3 lines, 18%). The most common *RET* fusions in each subgroup were *KIF5B-RET* (64% and 63%, respectively) and *CCDC6-RET* (18% and 22%, respectively).

### Treatment-Emergent Hypersensitivity Reactions With Selpercatinib

A total of 22 of 329 patients (7%) with *RET* fusion-positive NSCLC experienced a treatment-emergent AE reported using the Medical Dictionary for Regulatory Activities preferred term of “hypersensitivity” or “drug hypersensitivity” and attributed to selpercatinib by investigators (Table 2). Most patients (59%) experienced events grade 1 and 2. No life-threatening/debilitating or fatal events occurred. The median time to first onset of drug hypersensitivity was 1.7 weeks (range: 0.9–5.4 wk), and the median time to event resolution was 7 days (range: 3–34 d). Most of the patients who experienced a treatment-emergent hypersensitivity reaction had recovered (n = 20) or were recovering (n = 1) at the time of data cutoff; recovery had not yet been reported in one patient. There were two (1.3%) cases of pneumonitis in the ICI-treated group and 1 (0.6%) case of pneumonitis in the ICI-naive group. None of these events occurred in patients who experienced hypersensitivity reactions. More patients experienced a hypersensitivity reaction in the prior ICI-treated subgroup than in the ICI-naive subgroup (17 of 22, 77% versus 5 of 22, 23%) (Table 2). Events were reported as serious in eight (42%) prior ICI-treated patients and four (67%) ICI-naive patients. Median time to first onset of hypersensitivity was 1.7 weeks (range: 1.0–3.3 wk) in prior ICI-treated patients. The duration of time to the first hypersensitivity event from the patient’s last dose of prior ICI treatment was more than or equal to 1 to 2 months in 7 patients (41%), more than or equal to 2 to 3 months in 7 patients (41%), more than or equal to 3 to 6 months in two patients (12%), and more than or equal to 6 to 12 months in one patient (6%). The median time to first onset of hypersensitivity in patients naive to ICI therapy was 1.4 weeks (range: 0.9–5.4 wk).

**Figure 1.**
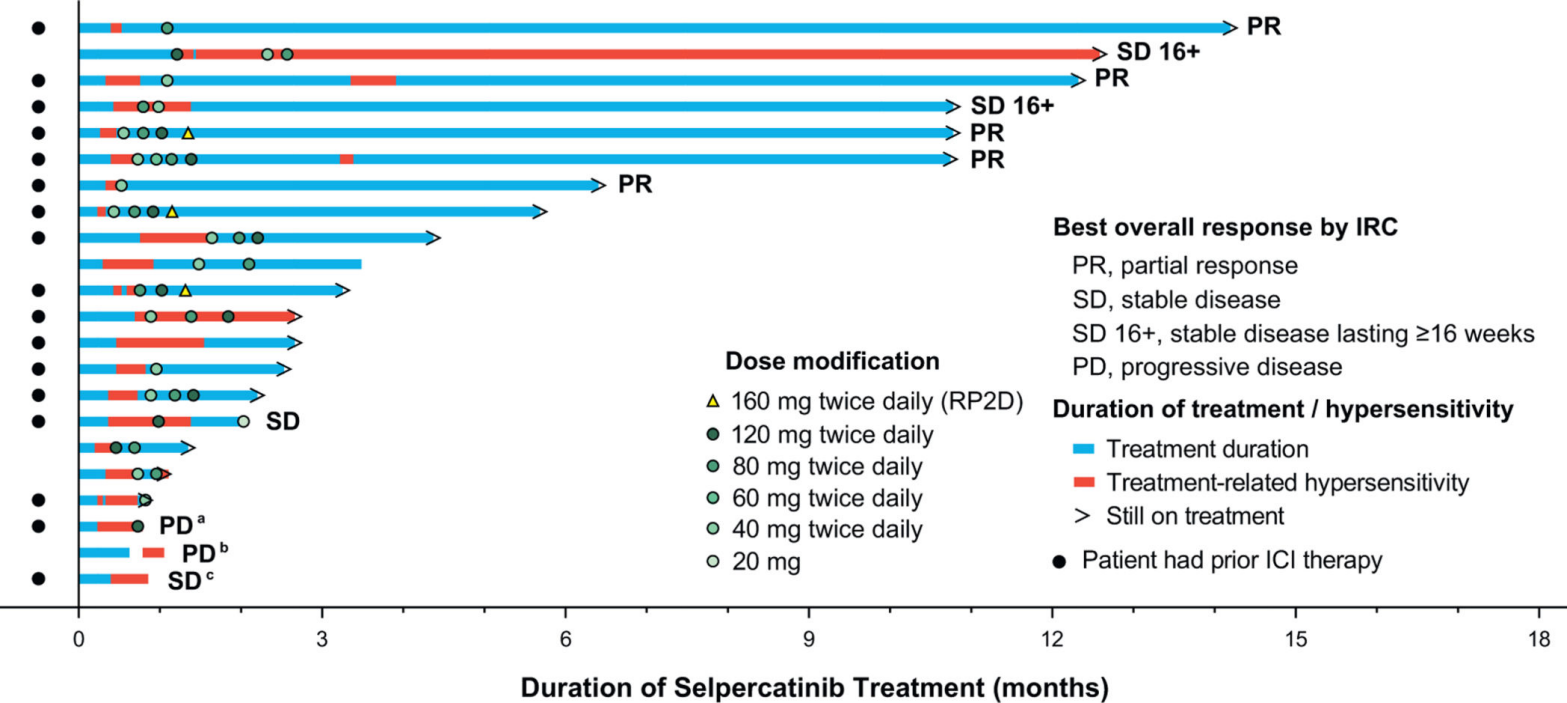
Treatment duration, dose modifications, and best overall response assessed by IRC with selpercatinib in patients with *RET* fusion-positive NSCLC who experienced a drug-related hypersensitivity reaction (N = 22). All patients began selpercatinib treatment on the recommended phase 2 dose of 160 mg twice daily. Best overall responses assessed by the IRC by data cutoff date of December 16, 2019, are presented. ^a^The patient permanently discontinued treatment with the first recurrence of hypersensitivity (grade 2) on rechallenge with a dose of 120 mg. ^b^The patient permanently discontinued treatment with the first event of hypersensitivity (grade 3) diagnosed 5 days after selpercatinib was discontinued. ^c^The patient permanently discontinued treatment with the first event of hypersensitivity (grade 2). ICI, immune checkpoint inhibitor; IRC, independent review committee; RP2D, recommended phase 2 dose.

Hypersensitivity reactions representative of the defined clinical constellation of events were attributed to selpercatinib treatment by investigators in 22 patients, of whom 17 had previously received ICI therapy (Fig. 1 and Table 2). Two patients permanently discontinued treatment with the first event of selpercatinib-related hypersensitivity (one grade 2 event in a prior ICI-treated patient and one grade 3 event in an ICI-naive patient). One patient who received prior ICI therapy permanently discontinued selpercatinib treatment with the first recurrence of grade 2 hypersensitivity upon rechallenge with a dose of 120 mg.

**Figure 2.**
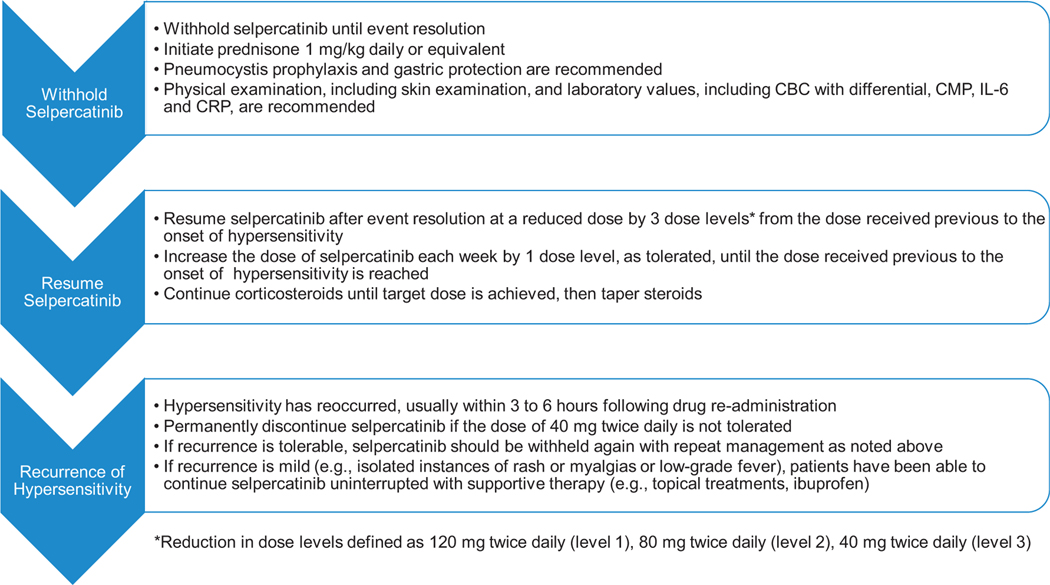
Guidelines for the management of selpercatinib-induced drug hypersensitivity. CBC, complete blood count; CMP, comprehensive metabolic panel; IL-6, interleukin-6; CRP, C-reactive protein.

Of the 22 patients with treatment-related hypersensitivity, 19 resumed selpercatinib with concurrent management strategies, including pretreatment with a steroid, graded rechallenge, and subsequent steroid taper, as outlined in Figure 2. No anaphylaxis or fatal outcomes occurred with selpercatinib rechallenge. All 19 patients had at least one dose reduction from their starting dose of 160 mg twice daily, and 14 of these patients received 40 mg twice daily as their lowest dose before sequential dose escalation to 80 mg twice daily, 120 mg twice daily, and 160 mg twice daily, as tolerated. Furthermore, 17 patients were still on treatment at the time of data cutoff and were taking doses of 40 mg twice daily (n = 5), 80 mg twice daily (n = 4), 120 mg twice daily (n = 4), and 160 mg twice daily (n = 4), on the basis of tolerability and response (Fig. 1). Among these patients, 14 (82%) had received prior ICI therapy.

**Figure 3.**
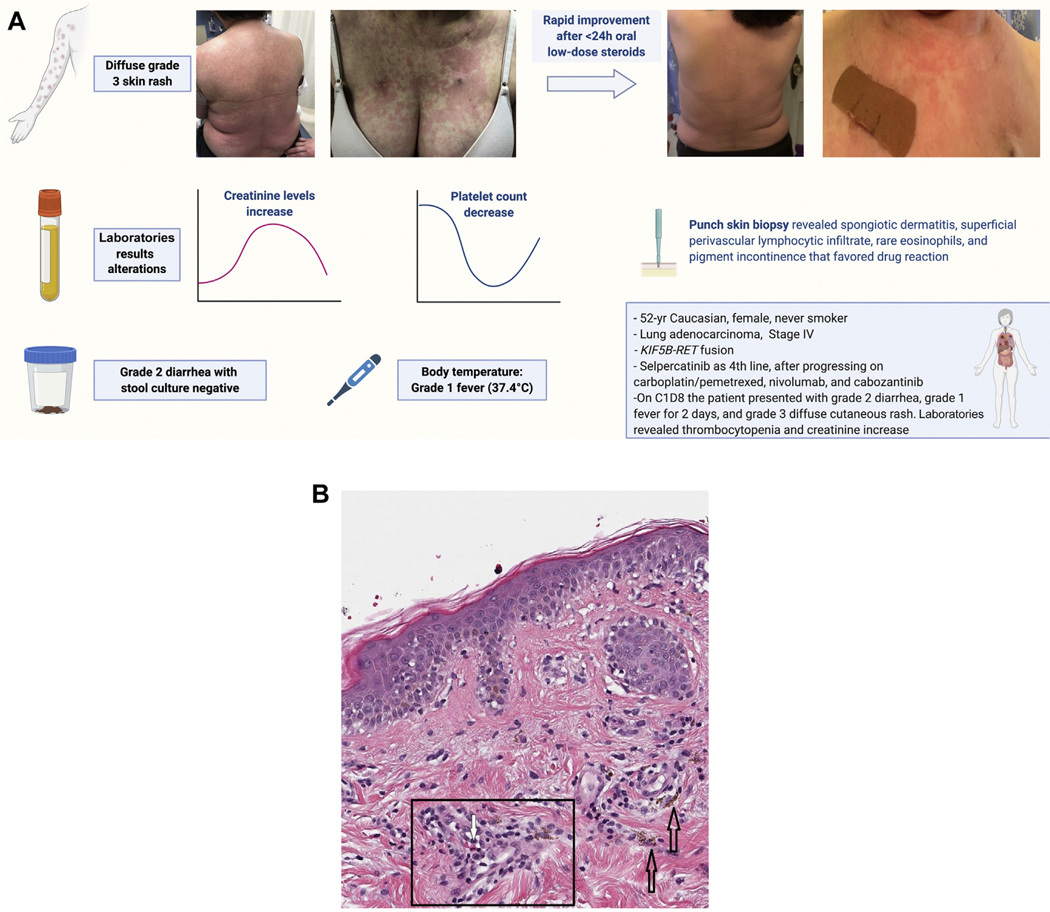
(*A*) An illustrative example of selpercatinib hypersensitivity. A 52-year-old white female, never smoker, with a stage IV NSCLC harboring a *KIF5B-RET* fusion. Selpercatinib was initiated as fourth-line therapy about 5 months after ICI therapy was discontinued. On C1D8 of selpercatinib 160 mg twice daily, she presented with grade 2 diarrhea (ultimately, results were negative for both *Clostridium difficile* and complete stool cultures), grade 1 fever (temperature of 37.4^◦^C) for 2 days, and grade 3 diffuse cutaneous rash. She was treated with ibuprofen, acetaminophen, and topical steroids, and the fever resolved. She also underwent punch skin biopsy (*B*), which revealed spongiotic dermatitis, superficial perivascular lymphocytic infiltrate, rare eosinophils, and pigment incontinence that favored drug reaction. Laboratory results revealed thrombocytopenia (nadir of 60th/*μ*L[60 × 10^9^/liter]) and elevated creatinine (up to 2.07 mg/dL [182.99 *μ*mol/liter]). Selpercatinib was held and prednisone 60 mg daily was initiated. Diarrhea and rash resolved after 48 h. Selpercatinib was resumed at 40 mg twice daily on C1D22 while continuing steroid therapy. The dose was sequentially escalated weekly to 80 mg twice daily, 120 mg twice daily, and then to the original starting dose of 160 mg twice daily. The patient achieved a partial response by RECIST 1.1 for 19 cycles of selpercatinib before developing progressive disease. Image was created with Biorender.com. (*B*) Skin pathology of selpercatinib hypersensitivity reaction. Histology of the erythematous eruption on the right side of the chest depicts skin with diffuse epidermal spongiosis/edema, perivascular lymphocytic inflammation (example within black box), and pigment incontinence (black arrows). Rare eosinophils are identified (white arrow). Image courtesy of Linda Huijie Song, MD, MSPH; University of Maryland, Department of Pathology. C1D22, cycle 1 day 22; CID8, cycle 1 day 8; h, hour; ICI, immune checkpoint inhibitor; RECIST 1.1, Response Evaluation Criteria in Solid Tumors version 1.1.

An illustrative example of a hypersensitivity reaction to selpercatinib in a prior ICI-treated patient is provided in Figure 3A and B. This patient case summarizes the common clinical features and abnormalities in laboratory results associated with this reaction and the management of these events with interruption and dose modification of selpercatinib and supportive care.

### Comparison of Overall Treatment-Emergent AEs With Selpercatinib Between Prior ICI and ICI-Naive Patients

Treatment-emergent AEs reported in 20% or more patients with *RET* fusion-positive NSCLC were largely grade 1 or 2 regardless of prior treatment with ICI (Table 3). The most common AEs of grade 3 or higher in the overall safety population were hypertension (17%), increased ALT (11%), increased AST (9%), and thrombocytopenia (5%). In prior ICI-treated and ICI-naive subgroups, these grade greater than or equal to 3 events occurred in 15% and 18% (hypertension), 14% and 9% (increased ALT), 12% and 5% (increased AST), and 6% and 4% (thrombocytopenia) of patients, respectively.

### Tumor Response With Selpercatinib in Relation to Hypersensitivity Reactions

Among the first 105 consecutively enrolled patients with *RET* fusion-positive NSCLC who were previously treated with at least platinum-based chemotherapy, 58 had received prior ICI therapy and 47 had no prior ICI therapy (Supplementary Table 1). The ORRs were similar between the prior ICI-treated and ICI-naive subgroups: 66% (95% confidence interval [CI]: 51.9–77.5) and 62% (95% CI: 46.4–75.5), respectively. The best overall response by IRC for prior ICI-treated and ICI-naive subgroups, respectively, was complete response (one patient in each subgroup), partial response (37 and 28 patients), stable disease (13 and 17 patients), and progressive disease (three and one patients). Median DoR was not reached in prior ICI-treated patients at a median follow-up of 12 months. For patients naive to ICI treatment, median DoR was 18 months (95% CI: 10.3–not evaluable) at a median follow-up of 13 months.

Among the 22 patients who experienced a drug hypersensitivity reaction related to selpercatinib treatment, 11 had been evaluated by IRC using Response Evaluation Criteria in Solid Tumors version 1.1 by the data cutoff date (Fig. 1). Of these patients, five had a confirmed partial response; four had stable disease, of whom two had stable disease lasting 16 weeks or more; and two patients had progressive disease. All patients with confirmed partial response had received prior ICI therapy and were still on selpercatinib treatment after one or more hypersensitivity reactions at doses of 40 mg twice daily (n = 2), 80 mg twice daily (n = 1), or 120 mg twice daily (n = 2).

## Discussion

Hypersensitivity is a known but uncommon AE associated with the administration of kinase inhibitors, including selpercatinib. Signs and symptoms associated with selpercatinib-induced hypersensitivity reactions were generally tolerable (i.e., maculopapular rash, fever, or arthralgias/myalgias associated with thrombocytopenia, increased AST or ALT, hypotension, tachycardia, or increased creatinine) and occurred more frequently in patients with *RET* fusion-positive NSCLC previously treated with ICI therapy. Though previous treatment with ICI therapy may increase the likelihood of hypersensitivity reactions with subsequent selpercatinib treatment, the overall safety and tolerability in this patient population was not substantially affected and most patients were able to remain on therapy with a strategy of steroid pretreatment, graded rechallenge, and subsequent steroid taper. Although not the focus of this report, it is important to note that rare (<1%) hypersensitivity reactions to selpercatinib treatment have been observed in patients with *RET*-altered thyroid cancers, revealing these events are not specific to NSCLC. The presentation and management of drug hypersensitivity did not change with tumor type.

After the implementation of the above-mentioned management strategies, all patients who attempted to re-initiate selpercatinib treatment after a reported drug-related hypersensitivity reaction were able to subsequently continue and tolerate the therapy. In most cases, selpercatinib treatment was continued at doses lower than the recommended starting dose of 160 mg twice daily, including 40 mg twice daily. Guidelines for investigators on the management of drug hypersensitivity reactions were developed and included in the IB and prescribing information (Fig. 2).

The rash and constellation of associated symptoms and abnormalities in laboratory results that are present with selpercatinib hypersensitivity reactions seem to have some similarities to other reported hypersensitivity reactions with consecutive administration of ICIs and kinase inhibitors and share similar outcomes of improvement on drug hold with steroid treatment.^10,11,13,14^ Although the exact mechanism of these hypersensitivity reactions on selpercatinib is unclear, they were deemed to be immune-mediated responses on the basis of the timing of onset and constellation of symptoms and findings. Owing to the variation in local investigative and treatment strategies, initial cases that informed the management strategy did not have uniformly available immune laboratory results (e.g., erythrocyte sedimentation rate, C-reactive protein, tryptase, leukotriene E4, interleukins [IL-2, IL-6, and IL-10], tumor necrosis factor, interferon gamma, eosinophils, immunoglobulin G, and histamine). Rather, a collective review of all available information across the patients was used to inform an understanding of the possible nature of these events and, therefore, to determine the proposed management strategy. Although the initial events had features compatible with a type IV immune-mediated hypersensitivity reaction, such as Drug Reaction with Eosinophilia and Systemic Symptoms (DRESS), they did not meet the comprehensive definition. For example, eosinophil levels were noted to be within normal limits and decreased from baseline, including lack of clinical features of DRESS, such as lymphadenopathy. Hypersensitivity reactions with selpercatinib treatment also had a shorter median time to onset compared with DRESS (1.7 wk versus 2 to 6 wk for DRESS), including a shorter median time to resolution (8 d versus 6–9 wk for DRESS). In addition, clinical and laboratory features of the reaction from the initial cases were noted (such as increased IL-6 levels), which were possibly consistent with cytokine-mediated reactions found in patients receiving targeted therapies after ICI.^13^

The higher rate of hypersensitivity in patients who received prior ICI therapy should give health care professionals pause when starting immunotherapy before understanding the tumor’s genetic makeup. Coupled with the efficacy found in LIBRETTO-001 and supported by a recent analysis revealing better efficacy outcomes with selpercatinib when compared with preenrollment therapies, including ICI,^17^ these findings highlight the importance of proactively screening patients to provide them with the best possible therapy options.

Lower efficacy outcomes have been observed with prior ICI therapy in patients with NSCLC and actionable driver alterations.^17–20^ Treatment guidelines for patients with NSCLC suggest initiating systemic treatment with targeted therapy, including *RET.*^21^ Nevertheless, limited access to next-generation sequencing assays or long turnaround times for results in clinical practice may lead to patients receiving nontargeted treatment.^22,23^ Despite the seemingly superior ORR to selpercatinib in patients with *RET* fusion-positive NSCLC who were naive to systemic treatment compared with their previously treated counterparts (85% versus 64%, respectively),^5^ clinically meaningful and durable responses were still observed regardless of prior ICI therapy and were consistent with the ORR in the overall previously treated cohort. These findings support the use of selpercatinib after ICI therapy regardless of the potential increased risk for hypersensitivity.

This analysis has some limitations. As AEs of “drug hypersensitivity” or “hypersensitivity” were reported, attempts were made to query the study site to verify whether these events met the criteria for the drug hypersensitivity analyzed; however, nonserious events were not reported with the same level of detail and, therefore, verification of the specific symptoms and findings may have been compromised. The multiple clinical factors that met the definition of drug hypersensitivity and spectrum of severity could have affected the threshold for identifying hypersensitivity reactions in this cohort and thus an underestimate of its frequency. In addition, given that this was not a defined physiological process, severity grading varied and was typically on the basis of a singular feature (e.g., rash, laboratory abnormality), rather than the collective findings. Differences in the severity of drug hypersensitivity reactions between prior ICI-treated and ICI-naive patients were therefore difficult to determine, as there was no predefined severity grade for these events. Because hypersensitivity reactions occurred in response to other drugs (e.g., antibiotics) or other factors (e.g., seasonal allergies), the frequency of hypersensitivity was reliant on events assessed by the investigators as related to selpercatinib treatment. Furthermore, it was not possible to determine whether the length of time between ICI therapy and selpercatinib treatment affected the occurrence or severity of the hypersensitivity response owing to reporting limitations, particularly given the prolonged half-life of ICI therapy. These differences, although potentially attributable to prior ICI therapy, may also be attributed to other factors such as disease state or additional prior lines of treatment. Studies to evaluate the underlying cause of drug hypersensitivity reactions with selpercatinib treatment were not required by protocol. Extensive panels for testing were both costly and varied in availability across study sites and were therefore not consistently done. In addition, tests performed that may have clarified the mechanism underlying these events were not always obtained at an ideal time point (e.g., cytokine levels drawn days after an event).

In conclusion, these data suggest that despite the higher incidence of treatment-related hypersensitivity reactions with selpercatinib observed in prior ICI-treated patients compared with ICI-naive patients, selpercatinib can be safely administered regardless of prior ICI status. Close monitoring for this serious AE is important for timely identification and treatment, as hypersensitivity reactions to selpercatinib can be managed with dose modification and supportive care measures and are reversible. In addition, hypersensitivity events do not seem to affect the overall efficacy of selpercatinib. The results of this analysis also suggest that consideration for sequencing of therapy should include comprehensive molecular testing at diagnosis to guide selection of the most appropriate systemic therapy. Similar to other kinase inhibitors, upfront ICI therapy in RET-altered patients may have implications, including increased frequency or severity of immune-mediated toxicities.

## Supplementary Material

1

## Figures and Tables

**Table 1.** Baseline Characteristics of Patients With *RET* Fusion-Positive NSCLC in the Overall Safety Population and in Subgroups With or Without Prior ICI Therapy

Characteristics	Prior ICI Therapy (n = 152)	No Prior ICI Therapy (n = 177)	Overall (N = 329)
Age, median (range), y	59 (26–80)	62 (23–92)	61 (23–92)
Sex, n (%)			
Female	79 (52.0)	106 (59.9)	185 (56.2)
Male	73 (48.0)	71 (40.1)	144 (43.8)
Race, n (%)			
White	77 (50.7)	88 (49.7)	165 (50.2)
Asian	66 (43.4)	70 (39.5)	136 (41.3)
Black or African American	5 (3.3)	11 (6.2)	16 (4.9)
Other or unknown	4 (2.6)	8 (4.5)	12 (3.6)
Prior cancer treatment, n (%)			
Number of systemic regimens			269 (81.8)
0	0	60 (33.9)	60 (18.2)
1–2	72 (47.4)	85 (48.0)	157 (47.8)
≥3	80 (52.6)	32 (18.1)	112 (34.0)
ICI therapy^a^	152 (100.0)	0	152 (46.2)
ECOG performance status, n (%)			
0	53 (34.9)	68 (38.4)	121 (36.8)
1	93 (61.2)	104 (58.8)	197 (59.9)
2	6 (3.9)	5 (2.8)	11 (3.3)
*RET* fusion, n (%)			
*KIF5B-RET*	97 (63.8)	111 (62.7)	208 (63.2)
*CCDC6-RET*	28 (18.4)	39 (22.0)	67 (20.4)
*NCOA4-RET*	5 (3.3)	3 (1.7)	8 (2.4)
Other	10 (6.6)	8 (4.5)	18 (5.5)
Unknown	12 (7.9)	16 (9.0)	28 (8.5)

*Note*: Data cutoff date of December 16, 2019.

a Prior ICI therapies were atezolizumab, avelumab, cemiplimab, durvalumab, nivolumab, pembrolizumab, and spartalizumab. ECOG, Eastern Cooperative Oncology Group; ICI, immune checkpoint inhibitor.

**Table 2.** Hypersensitivity Reactions Reported As Selpercatinib-Related in Patients With *RET* Fusion-Positive NSCLC in the Overall Safety Population and in Subgroups With or Without Prior ICI Therapy

AE Characteristics	Prior ICI Therapy (n = 152)	No Prior ICI Therapy (n = 177)	Overall (N = 329)
TEAE of hypersensitivity, n (%)	17 (11.2)	5 (2.8)^a^	22(6.7)
Highest severity			
Grade 1	1 (0.7)	0	1(0.3)
Grade 2	11 (7.2)	1 (0.6)	12 (3.6)
Grade 3	5 (3.3)	4 (2.3)	9 (2.7)
Grade 4	0	0	0
Grade 5	0	0	0
Serious AE	8 (5.3)	4 (2.3)	12 (3.6)
Related to selpercatinib	17 (11.2)	5 (2.8)	22 (6.7)
Serious-related AE	8 (5.3)	4 (2.3)	12 (3.6)
Dose modification^b^			
Interruption	4 (2.6)	2 (1.1)	6 (1.8)
Reduction	14 (9.2)	4 (2.3)	18 (5.5)
Discontinuation	2 (1.3)	1 (0.6)	3 (0.9)
Time to first onset			
Median (range), wk	1.7 (1.0–3.3)	1.4 (0.9–5.4)	1.7 (0.9–5.4)
Outcome of last episode			
Recovered/resolved	16 (10.5)	4 (2.3)	20 (6.1)
Recovering/resolving	1 (0.7)	0	1 (0.3)
Not recovered/not resolved	0	1 (0.6)	1 (0.3)

*Note:* Data cutoff date of December 16, 2019. This analysis was based on consolidated Medical Dictionary for Regulatory Activities (version 21.0) preferred terms of “hypersensitivity” and “drug hypersensitivity.” Results are presented regardless of reported causality unless otherwise specified. Patients with multiple severity ratings for one or more hypersensitivity events were counted once under the maximum severity. Assignment of severity grade was based on Common Terminology Criteria for Adverse Events (version 4.03): grade 1 (mild), grade 2 (moderate), grade 3 (severe), grade 4 (life threatening/debilitating), and grade 5 (fatal). A grade 1 or 2 event considered by the investigator to be medically important was reported as a serious event.

a Patients in the “no prior ICI” category were either previously treated with chemotherapy without ICI (117) or naive to prior treatment (60); three of the five TEAEs of hypersensitivity in this subgroup occurred in treatment-naive patients.

b For each hypersensitivity event resulting in dose modification, a patient was counted once under the most substantial action taken (discontinuation, reduction, or interruption, respectively) if more than one action occurred. A patient who experienced multiple events could be counted under more than one action.

AE, adverse event; ICI, immune checkpoint inhibitor; TEAE, treatment-emergent adverse event.

**Table 3.** Adverse Events Reported in Greater Than or Equal to 20% of Selpercatinib-Treated Patients With RET Fusion-Positive NSCLC in the Overall Safety Population and in Subgroups With or Without Prior ICI Therapy

AEs	Prior ICI Therapy (n = 152)		No Prior ICI Therapy (n = 177)		Overall (N = 329)	
n (%)	Any Grade	Grade ≥ 3	Any Grade	Grade ≥ 3	Any Grade	Grade ≥ 3
Any TEAE	150 (98.7)	96 (63.2)	175 (98.9)	100 (56.5)	325 (98.8)	196 (59.6)
Dry mouth	57 (37.5)	0	77 (43.5)	0	134 (40.7)	0
Diarrhea	56 (36.8)	5 (3.3)	77 (43.5)	4 (2.3)	133 (40.4)	9 (2.7)
Increased ALT	50 (32.9)	21 (13.8)	52 (29.4)	16 (9.0)	102 (31.0)	37 (11.2)
Increased AST	50 (32.9)	19 (12.5)	58 (32.8)	9 (5.1)	108 (32.8)	28 (8.5)
Hypertension	47 (30.9)	23 (15.1)	58 (32.8)	32 (18.1)	105 (31.9)	55 (16.7)
Peripheral edema	40 (26.3)	0	41 (23.2)	0	81 (24.6)	0
Fatigue	37 (24.3)	3 (2.0)	41 (23.2)	1 (0.6)	78 (23.7)	4 (1.2)
Pyrexia	37 (24.3)	1 (0.7)	28 (15.8)	0	65 (19.8)	1 (0.3)
Thrombocytopenia	36 (23.7)	9 (5.9)	21 (11.9)	7 (4.0)	57 (17.3)	16 (4.9)
Nausea	32 (21.1)	1 (0.7)	37 (20.9)	1 (0.6)	69 (21.0)	2 (0.6)
Rash	32 (21.1)	0	39 (22.0)	2 (1.1)	71 (21.6)	2 (0.6)
Headache	31 (20.4)	1 (0.7)	34 (19.2)	2 (1.1)	65 (19.8)	3 (0.9)
Constipation	26 (17.1)	0	40 (22.6)	3 (1.7)	66 (20.1)	3 (0.9)

*Note:* Data cutoff date of December 16, 2019.

ALT, alanine aminotransferase; AST, aspartate aminotransferase; ICI, immune checkpoint inhibitor; TEAE, treatment-emergent adverse event.
