# Supplementary material for: Hypersensitivity Reactions to Selpercatinib Treatment With or Without Prior Immune Checkpoint Inhibitor Therapy in Patients With NSCLC in LIBRETTO-001
Source: J Thorac Oncol. Author manuscript; Available in PMC 2024 May 10. (PMC11083849; doi:10.1016/j.jtho.2022.02.004)
Supplement: 1 [file NIHMS1862128-supplement-1.docx]

**Table S1.** Response to selpercatinib treatment in patients with *RET* fusion-positive NSCLC who were previously treated with platinum-based chemotherapy (N=105), with or without prior ICI therapy

|  | **Prior ICI Therapy**  **(n=58)** | **No Prior ICI Therapy**  **(n=47)** |
| --- | --- | --- |
| **ORR by IRC, % (95% CI)** | 66 (51.9–77.5) | 62 (46.4–75.5) |
| **Best overall response, n (%)** | | |
| Complete response | 1 (1.7) | 1 (2.1) |
| Partial response | 37 (63.8) | 28 (59.6) |
| Stable disease | 13 (22.4) | 17 (36.2) |
| Progressive disease | 3 (5.2) | 1 (2.1) |
| Not evaluable | 4 (6.9) | 0 |
| **Duration of response** |  |  |
| Median DoR, months (95% CI) | NE (12.0–NE) | 17.5 (10.3–NE) |
| Median follow-up, months | 11.9 | 12.7 |
| Data cutoff date of 16 December 2019.  Abbreviations: DoR, duration of response; ICI, immune checkpoint inhibitor; IRC, independent review committee; NE, not evaluable; ORR, objective response rate. | | |
